# Unveiling Intersecting Experiences: Investigating Health Care and Jail System Interaction Before and After Incarceration Among Adults with Serious Mental Illness in San Francisco

**DOI:** 10.1007/s11524-026-01058-2

**Published:** 2026-02-24

**Authors:** Meghan Hewlett, Eve Perry, Dave Graham-Squire, Alissa Skog, Johanna Lacoe, Hemal Kanzaria, Jacob Izenberg, Maria Raven

**Affiliations:** 1 Department of Emergency Medicine, University of California, San Francisco, CA USA; 2 Benioff Homelessness and Housing Initiative, University of California, San Francisco, CA USA; 3 California Policy Lab, University of California, Berkeley, CA USA; 4 Department of Psychiatry and Behavioral Sciences, University of California, San Francisco, CA USA

**Keywords:** Health services, Incarceration, Mental illness

## Abstract

Individuals with serious mental illness (SMI) are overrepresented in the criminal justice system. They frequently cycle between jail and emergency departments and experience poor health outcomes. Some jails offer mental health services, but the criminal justice environment is not conducive to optimal treatment and recovery. Individuals with SMI may require targeted interventions, but few studies have examined characteristics of individuals in jail with and without an SMI or compared their post-release trajectories. We conducted a retrospective cross-sectional study using health services and jail system data from the City and County of San Francisco from July 1, 2011 to June 30, 2021. We included 1568 adults with at least one jail incarceration from July 1, 2017 to June 30, 2018, stratified by the presence or absence of an SMI diagnosis, and compared health services use, jail system interactions, and associated factors. Compared to individuals without SMI, those with SMI had higher rates of substance use disorder (82.5% vs. 53.3%) and homelessness (82.2% vs. 61.0%). The longest single jail incarceration averaged 11.3 days longer for individuals with SMI (*p* < 0.05, 95% CI: 1.38, 21.26). Post-release, individuals with SMI had higher rates of health services use and reincarceration. We found that individuals with SMI in jail have higher rates of homelessness, health services use, and jail reincarceration. This underlines the responsibility placed on jails to address the complex needs of individuals with SMI. Investment should focus on reentry and diversion programs, as well as expanding jail and community-based mental health care for individuals with SMI.

**Supplementary Information:**

The online version contains supplementary material available at 10.1007/s11524-026-01058-2.

## Introduction

Adults in the United States living with serious mental illness (SMI), such as schizophrenia, bipolar disorder, and major depressive disorder, must navigate an under-resourced treatment landscape that lacks adequate capacity to provide low-barrier access for high-need patients such as those with co-occurring substance use disorder and homelessness [1]. Relative to those without SMI, adults with SMI have worse physical health status and disproportionately higher rates of urgent/emergent service use—indicating unmet health and social needs [2, 3]. This vulnerability is heightened by a higher risk for incarceration among those with SMI, which creates further barriers to accessing and adhering to outpatient mental health treatment and resources.

Individuals with SMI comprise approximately 20% and 43% of the United States (US) population who are incarcerated and experiencing homelessness, respectively [4–6]. Furthermore, adults with SMI are ten times more likely to be incarcerated than to receive adequate mental health treatment [7–10]. Compared to individuals with a single jail arrest in a year, those with multiple arrests are more likely to have SMI, experience psychiatric crises, have a co-occurring substance use disorder (SUD), lack health insurance, and frequently use emergency department (ED) services [11]. The relationship between SMI, homelessness, and incarceration is complex and derives from structural and systemic inequities embedded within criminal justice, health care, housing, and employment systems which, in turn, significantly influence health [12]. Despite the frequency of jail incarceration among individuals with SMI and the knowledge that such incarcerations can disrupt an already fragmented pattern of care seeking, this population has not been well described in the literature or compared to individuals without SMI who are also jail incarcerated. A better understanding of the unique health and social care needs of individuals with SMI incarcerated in jail could inform policies and interventions that enhance both in-jail treatment and post-release care. To address this knowledge gap, we describe and compare patterns of health care service use and jail system interaction between individuals with and without SMI before, the year of, and after jail incarceration. We additionally describe housing status, prior jail incarceration histories, and physical health statuses.

While much of the literature around incarceration and health focuses on prisons, jail data are relevant for individuals with SMI for several reasons. Jails incarcerate individuals awaiting trial or serving short sentences, resulting in high turnover rates, and therefore account for individuals with multiple booking incidents within a year. Conversely, prisons incarcerate individuals for longer durations, leading to more stable populations. Jail incarceration also does not necessarily indicate that an individual has been found guilty of a crime, as many people detained are awaiting trial; thus, it is not a perfect proxy for criminal behavior. Frequent cycling in and out of jail is pertinent to this study, as individuals with multiple jail bookings within a year are more likely to have SMI and frequently use emergency department services [11]. Analyzed in aggregate, high jail turnover rates may also interrupt outpatient health care receipt for individuals with complex health care services needs such as those with SMI. Furthermore, health issues prevalent within jail populations have broad public health implications, given the rapid movement of individuals between the community and the jail system.

## Methods

### Study Design and Setting

We conducted a retrospective cross-sectional study of adults in the City and County of San Francisco using a de-identified registry that links physical health, behavioral health, and social services data from the San Francisco Department of Public Health’s (SFDPH) Coordinated Care Management System (CCMS) with county jail system data from San Francisco Sheriff’s office for fiscal years 2011–2021 (July 1, 2011–June 30, 2021). The CCMS has been described in detail elsewhere [13–15]. Briefly, the CCMS integrates information about complex, high-needs patients across multiple service domains by combining data from several county agencies and the San Francisco Health Plan (SFHP), San Francisco County’s primary Medicaid managed care plan. The CCMS includes medical and behavioral electronic health care records, homelessness services, and jail health encounters. The CCMS creates a record for any patient (a) reported as unhoused by a San Francisco County agency, or (b) with county jail contact, or (c) who uses urgent or emergent county medical, MH, or SUD services (SupplementalTable 1**)**. The database integrates data that can be matched at the patient level.

**Table 1:** Comparison of pre-incarceration period demographics, health care and county jail system interaction between individuals with and without serious mental illness

Demographics	Total *N* = 1568	With SMI No. (%)*n* = 732 (46.7%)	Without SMI No. (%) *n* = 836 (53.3%)	*p*-value^a^
Age, mean, (± SD) (years)		40.8 (11.2)	40.1 (11.5)	0.2
18–24		38 (5.2%)	71 (8.5%)	
25–34		204 (27.9%)	232 (27.8%)	
35–44		219 (29.9%)	231 (27.6%)	
45–54		175 (23.9%)	192 (23.0%)	
55–64		81 (11.1%)	96 (11.5%)	
65 +		15 (2.0%)	14 (1.7%)	
Gender				
Men		568 (77.6%)	656 (78.5%)	0.5
Race and ethnicity				0.027
Asian or Pacific Islander		38 (5.2%)	32 (3.8%)	
Black		294 (40.2%)	396 (47.4%)	
Latinx		104 (14.2%)	118 (14.1%)	
Multi-racial and other^b^		36 (4.9%)	27 (3.2%)	
White		260 (35.5%)	263 (31.5%)	
Housing status				
Reported within two prior FYs		730 (99.7%)	813 (97.2%)	
Evidence of homelessness				< 0.001
Within two prior FYs		602 (82.2%)		
Before two prior FYs		33 (4.5%)	64 (7.7%)	
Never		97 (13.3%)	262 (31.3%)	
Health status				
Elixhauser physical health comorbidity score, median (IQR)		3.0 (2.0–4.0)	1.0 (0.0–2.0)	< 0.001
SUD diagnosis				< 0.001
Within two prior FYs		604 (82.5%)	446 (53.3%)	
Before two prior FYs		47 (6.4%)	85 (10.2%)	
Never		81 (11.1%)	305 (36.5%)	
Health care services use^c^				
Urgent/emergent physical health services use, median, (IQR), (visits/admissions)		7.0 (4.0–16.0)	5.0 (3.0–9.0.0.0)	< 0.001
Urgent/emergent mental health services use, median, (IQR), (visits/admissions)		2.0 (0.0–2.0)	0.0 (0.0-0.0.0.0)	< 0.001
Urgent/emergent SUD services use, median, (IQR), (visits/admissions)		0.0 (0.0–1.0)	0.0 (0.0-0.0.0.0)	< 0.001
County jail system interaction				
History of prior jail booking		637 (87.0%)	723 (86.5%)	0.8
Cumulative jail days for prior incarcerations, median, (IQR), (Days)^d^		99.9 (9.7–344.5)	76.0 (8.2–328.0)	0.3

*FY*, fiscal year: *SMI*, serious mental illness; *SUD*, substance use disorder

Numbers may not sum to 100% due to rounding error

^a^Kruskall-Wallis rank sum test used for continuous variables, Pearson’s Chi-squared test used for categorical variables

^b^The Multiracial and Other category was combined due to small numbers among individuals self-identifying as Native American or Indigenous, Multiracial, and those not reporting a race or ethnicity

^c^Counts obtained from the 2015–2016 and 2016–2017 fiscal years

^d^Individuals with no prior booking history excluded

We linked CCMS with an additional criminal justice dataset comprised of jail booking data from the San Francisco Sheriff’s Office, including all people booked into the county jail on new criminal charges, pretrial violations, supervision violations, and out-of-county warrants. The linkage of the datasets has been previously described [13]. Briefly, we linked them via supervised machine learning using Python’s Dedupe (Forest Gregg and Derek Eder, 2022, Dedupe), using each individual’s first name, last name, and date of birth. Once all datasets were linked, we removed personal identifying information and created a new individual-level unique identifier. Data linkage, storage, and analyses were performed via a secure, HIPAA-compliant platform. Researchers trained and approved to work with Protected Health Information analyzed the data. The University of California San Francisco Institutional Review Board provided approval for this study. Reporting complies with STROBE guidelines [16].

### Study Population and Measures

We included all adults at least 18 years of age with at least one county jail incarceration related to a new charge during the 2017–2018 fiscal year (July 1, 2017–June 30, 2018; hereafter defined as the incarceration period) and a record in the CCMS for at least two fiscal years preceding the incarceration period (July 1, 2015 (or earlier)–June 30, 2017; hereafter defined as the pre-incarceration period).

Following criteria from a prior study [17], we defined a group of individuals as having SMI if they had two or more service encounters recorded in the CCMS with a principal diagnosis code (International Classification of Diseases, Ninth and Tenth Revision, Clinical Modification; ICD-9-CM, ICD-10-CM) in the psychosis or depression Elixhauser categories within the two fiscal years preceding the incarceration period. We designated all other individuals within the study population as not having SMI.

We calculated individual-level physical health comorbidity burdens by obtaining an unweighted sum of unique Elixhauser diagnosis categories corresponding to principal physical health diagnoses for associated health encounters during the fiscal year preceding the incarceration period (hereafter known as the Elixhauser Physical Health Comorbidity Score)(Supplemental Table 2**)**. We ascertained past and current homelessness through observed use of homelessness services and self-reported homelessness during service encounters [15].

**Table 2:** Comparison of county jail system interaction during the incarceration period (2017–2018 fiscal year) between individuals with and without serious mental illness

	With SMI No. (%) *n* = 732 (46.7%)	Without SMI No. (%) *n* = 836 (53.3%)	*p*-value^a^
Total number of jail bookings, median, (IQR)	1.0 (1.0–2.0)	1.0 (1.0–2.0)	0.043
Cumulative days incarcerated, median, (IQR)	5.9 (1.5–44.9)	4.0 (1.1–32.1)	0.003
Longest single incarceration, median, (IQR), (Days)	5.0 (1.4–38.7)	3.7 (1.1–23.5)	0.002

^a^Kruskall-Wallis rank sum test used for continuous variables

*FY*, fiscal year; *SMI*, serious mental illness; *SUD*, substance use disorder

For the pre-incarceration period we assessed individuals’ gender identity, housing status and housing services use; health status; urgent and emergent health services use within the two fiscal years preceding the incarceration period (as a proxy for difficulty accessing outpatient care) [18]; history of prior jail bookings; and cumulative jail days for all prior jail incarcerations. For the incarceration period, we analyzed individuals’ age at their first jail booking; jail booking data; cumulative jail days for all jail incarcerations during this period; and their longest single jail incarceration. We defined the 2018–2019 fiscal year (July 1, 2018–June 30, 2019) as the post-incarceration period, and if such data were present for an individual, we assessed the following: jail booking data for new and existing cases; housing status and housing service use; and urgent and emergent health services use.

### Statistical Analysis

We used descriptive statistics to compare the demographic characteristics and patterns of service use for people with and without SMI for each period (pre-incarceration, incarceration, and post-incarceration). We report frequencies for categorical variables and reported measures of central tendency and dispersion for continuous variables. We conducted an ordinary least-squares regression analysis to compare the length of the longest single jail stay during the incarceration period for those with and without SMI. The regression model included controls for age and gender. We did not include a control for a SUD diagnosis due to collinearity concerns resulting from the substantial overlap between SUD and SMI. To evaluate the statistical significance of the regression coefficients, we constructed 95% confidence intervals. We used R version 4.3.1 for all statistical analyses. We also conducted a sub-analysis of the individuals with SMI during the post-incarceration period, comparing jail booking prevalence based on whether they used urgent/emergent mental health services.

## Results

Of the 11,040 adults with at least one jail booking during the incarceration period, 1568 also had a record in CCMS (14.2%), resulting in a study population with 732 (46.7%) and 836 (53.3%) individuals comprising the SMI and non-SMI groups, respectively.

### Pre-Incarceration Period

#### Demographics and Housing Status

The mean (± SD) age was similar between individuals with and without SMI (40.8 (± 11.2) years and 40.1 (± 11.5) years, respectively) **(**Table 1**)**. Gender distribution was also similar between both groups. Housing status was recorded in our data within the two fiscal years prior to the incarceration period for 99.7% and 97.2% of individuals in the SMI and non-SMI groups, respectively. While both groups had a high prevalence of homelessness, the SMI group, compared to the non-SMI group, had a higher percentage of individuals experiencing homelessness in the pre-incarceration period (82.2% vs. 61.0%, respectively) and a lower percentage of individuals who had never experienced homelessness (13.3% vs. 31.3%, respectively).

#### Health Status and Urgent/Emergent Health Services Use

The SMI group had a higher median (IQR) Elixhauser Physical Health Comorbidity Score compared to the non-SMI group (3.0 (2.0–4.0) vs. 1.0 (0.0–2.0)). A higher Elixhauser score signifies a greater burden of chronic diseases and potentially increased health care needs. The SMI group also had a higher proportion of individuals who met criteria for a SUD diagnosis during the pre-incarceration period (82.5% vs. 53.5%). More individuals in the non-SMI group had never been diagnosed with a SUD (36.5% vs. 11.1%). Regarding urgent/emergent health services use during the pre-incarceration period, almost all individuals in both groups used physical health services, with individuals in the SMI group having a higher median (IQR) number of visits relative to individuals in the non-SMI group (7.0 (4.0–16.0) vs. 5.0 (3.0–9.0), respectively). The SMI group also had a higher median (IQR) number of mental health services visits compared to the non-SMI group (2.0 (0.0–2.0) vs. 0.0 (0.0–0.0), respectively).

#### County Jail System Interaction History

Individuals with and without SMI group were equally as likely to have a history of a jail booking during the pre-incarceration period (87.0% vs. 86.5%). Supplemental Fig. 1 shows the distribution of cumulative days spent in jail for all prior incarcerations by SMI status. Individuals with no prior jail booking history were not included in this figure. Among individuals with at least one prior incarceration, the cumulative jail days for all incarcerations over this period were similarly distributed for both groups; however, the median (IQR) cumulative jail days for the SMI group was almost 25% higher than the non-SMI group (99.9 (9.7–344.5) vs. 76.0 (8.2–328.0)).

### Incarceration Period

Supplemental Figs. 2 and 3 display the distribution of total jail bookings and cumulative jail days for all incarcerations, respectively, by SMI status. The total number of bookings during this period was similarly distributed between both groups, as was the cumulative number of jail days. Table 2 shows a comparison of county jail system interaction during this period between individuals with and without SMI, and Supplemental Fig. 4 displays the distribution of the longest single jail incarceration by SMI status. The SMI group had a higher median number (IQR) of cumulative jail days compared to the non-SMI group (5.9 (1.5–44.9) vs. 4.0 (1.1–32.1)), and a higher median (IQR) duration of the longest single incarceration at 5.0 days (1.4–38.7) compared to 3.7 days (1.1–23.5) for the non-SMI group.

#### Multivariate Analysis

In our multivariate analysis, we found that SMI was significantly associated with having a longer single jail stay: the longest jail stay was 11.1 days longer in the SMI group compared to the non-SMI group after adjustment (*p *< 0.05, 95% CI: 1.06, 21.14) **(**Table 3**)**.

**Table 3:** Multivariable analysis of the longest single jail stay during the incarceration period

Mental health status	Estimate (95% CI)
With SMI	11.09* (1.05, 21.13)
Without SMI	Reference

^*^p < 0.05; the model was adjusted for the following covariates: age, race and ethnicity, and gender

### Post-Incarceration Period

#### County Jail System Interaction

Both groups had similar percentages of individuals that had at least one jail booking during this period (49.6% among individuals with SMI and 46.4% among individuals without SMI) **(**Table 4**)**. However, individuals with SMI were more likely to have repeated bookings related to the same case compared to those without SMI (19.4% vs. 15.6%, respectively).

**Table 4:** Comparison of post-incarceration housing status, health care and county jail system interaction between individuals with and without serious mental illness

County jail system interaction	With SMI No. (%) (*N* = 732, 46.7%)	Without SMI No. (%) (*N* = 836, 53.3%)	*p*-value^a^
Jail booking for a new case	327 (44.7%)	354 (42.4%)	0.4
Jail booking for an open case	142 (19.4%)	130 (15.6%)	0.052
Any jail booking	363 (49.6%)	388 (46.5%)	0.2
Total number of jail bookings, Median, (IQR)	0.0 (0.0–2.0)	0.0 (0.0–2.0)	0.030
Housing status^b^			
Reported in the CCMS	510 (69.7%)	475 (56.8%)	< 0.001
Evidence of homelessness	401 (54.8%)	322 (38.5%)	< 0.001
Health care services use^c^			
Reported in the CCMS	535 (73.1%)	544 (65.1%)	< 0.001
Used urgent/emergent physical health services	494 (92.3%)	541 (99.4%)	< 0.001
Used urgent/emergent mental health services	278 (52.0%)	36 (6.6%)	< 0.001
Used urgent/emergent SUD services	62 (11.6%)	42 (7.7%)	0.040
	With mental health services use	No mental health services use	***p***-value^a^
County jail system interaction among individuals with SMI			
Jail booking for a new case	171 (61.5%)	113 (44.0%)	< 0.001
Jail booking for an open case	79 (28.4%)	45 (17.5%)	0.004
Any jail booking	187 (67.3%)	121 (47.1%)	< 0.001

Numbers may not sum to 100% due to rounding error

^a^Kruskall-Wallis test used for continuous variables, Pearson’s Chi-squared test used for categorical variables

^b^Individuals without a recorded housing status were presumed to not be experiencing homelessness. Individuals not in the CCMS were excluded from service use measures

*CCMS*, the Coordinated Care Management System; *SMI*, serious mental illness;* SUD*, substance use disorder

#### Housing Status

Housing status was reported in the data in the post-incarceration period for 69.7% and 56.8% of individuals in SMI and non-SMI groups, respectively. Of these groups for whom housing status was reported, 54.8% and 38.5% of individuals in SMI and non-SMI groups were reported to be experiencing homelessness.

#### Urgent/Emergent Health Services Use

Post incarceration, more individuals from the SMI group had service use data reported in the CCMS compared to the non-SMI group (73.1% vs. 65.1%, respectively). Among individuals for whom CCMS data was available, a smaller proportion of individuals in the SMI group used physical health services compared to the non-SMI group (92.3% vs. 99.4%, respectively). However, more individuals in the SMI group used mental health (52.4% vs. 6.4%) and SUD services (11.6% vs. 7.6%) compared to the non-SMI group.

#### Sub-analysis of Individuals with SMI

When we stratified the individuals in the SMI group by their use of urgent/emergent mental health services, we found a correlation between urgent/emergent mental health services use and jail bookings: individuals with any urgent/emergent mental health service use had higher proportions of new and repeat bookings compared to those without any service use (61.5% vs. 44.0%, 28.4% vs. 17.5%).

## Discussion

To our knowledge, this is the first study to examine differences in health service use, diagnoses, and housing status trajectories between jail incarcerated adults with and without SMI. Our study highlights the heightened challenges faced by individuals with SMI and criminal justice system involvement, including higher rates of co-occurring SUD, homelessness before and after jail incarceration, longer jail incarceration durations, higher rates of repeat jail bookings, and greater reliance on urgent and emergent health services post-incarceration. Individuals with SMI who used urgent/emergent mental health services during the post-incarceration period were more frequently re-booked in jail compared to those who did not use them, consistent with prior research linking fragmented mental health care to increased reincarceration rates [19]. These findings point to gaps in community mental health services, exacerbated by barriers such as inadequate care coordination, geographic disparities in mental health care services availability, and competing demands related to housing and SUD care. While California’s Medi-Cal program (the state’s Medicaid health care program) has expanded insurance coverage; access to outpatient mental health care remains hindered by these challenges [20].

Barriers to successful community reentry (e.g., homelessness) compound the risk of reincarceration for individuals with SMI. Criminalization of homelessness through “nuisance laws” further perpetuates cycles of instability, highlighting the need for supportive housing and social services to address upstream drivers of incarceration [21, 22]. While our results cannot demonstrate directionality, they support this finding [11, 23]. While jails are increasingly tasked with addressing the complex health care needs of individuals with SMI, which are unmet in the community, they are not designed or adequately funded to function as clinical treatment facilities [24–26].

In our multivariate analysis, we found that the longest jail stay during the incarceration period for individuals with SMI averaged approximately eleven days longer compared to those without SMI, after adjustment. We did not have access to court data and could not determine if those in our study were directed to behavioral health court (BHC) or other mental health diversion programs, which have been implemented widely across the United States as an alternative to criminal prosecution by providing individuals with mental illness with wraparound services, including mental health care services and case management. The Superior Court of California for the County of San Francisco has a BHC that monitors about 140 clients at a time; therefore, this longer incarceration duration for those with SMI may be attributable to treatment plan development and care coordination by the BHC [27].

Our findings are limited by the use of administrative data which may underreport service use, demographics, and outcomes. Our ascertainment of past and current homelessness through the use of homelessness services and self-reported homelessness during service encounters may underestimate the number of individuals in our study population previously or currently experiencing homelessness. Mortality during the post-incarceration period may explain some attrition, particularly given the elevated mortality rates among individuals experiencing homelessness [28]. Additionally, as this was a descriptive study, we did not employ propensity score matching to account for baseline differences, and results may not be generalizable beyond San Francisco. Other jurisdictions outside of San Francisco may enforce “nuisance laws” more rigorously, as well as their availability of diversion programs, which also limits our study’s generalizability. Despite these limitations, the study highlights systemic gaps in mental health care for individuals with SMI.

Our findings underscore the need for health policies that prioritize investment in community-based mental health services, housing support, and SUD care to reduce reliance on jails for mental health treatment [29, 30]. The California Advancing and Innovating Medi-Cal (CalAIM) initiative, a multi-year plan to improve the state’s Medicaid program including its integration with social services, is one example of addressing gaps in care [31]. Additionally, investments in jail health services and care coordination are necessary to improve outcomes during incarceration and facilitate successful reentry. Community-based diversion programs, such as behavioral health courts, and upstream interventions, including mobile crisis teams and crisis intervention training for law enforcement, can prevent criminal justice involvement and address systemic drivers of incarceration for individuals with SMI.

## Supplementary Information

Below is the link to the electronic supplementary material.

ESM 1 (DOCX 124 KB)

ESM 2 (DOCX 76.2 KB)

ESM 3 (DOCX 179 KB)

ESM 4 (DOCX 118 KB)

ESM 5 (DOCX 27.7 KB)

ESM 6 (DOCX 13.2 KB)

ESM 7 (DOCX 12.8 KB)
