# Supplementary material for: Unveiling Intersecting Experiences: Investigating Health Care and Jail System Interaction Before and After Incarceration Among Adults with Serious Mental Illness in San Francisco
Source: J Urban Health. 2026 Feb 24;103(3):533–41. doi: 10.1007/s11524-026-01058-2 (PMC13315379; doi:10.1007/s11524-026-01058-2)
Supplement: Supplementary file 1 — (DOCX 124 KB) [file 11524_2026_1058_MOESM1_ESM.docx]

**Supplemental Fig. 1 Pre-Incarceration Period: Distribution of Cumulative Jail Days for all Prior Jail Incarcerations by Serious Mental Illness Status.**


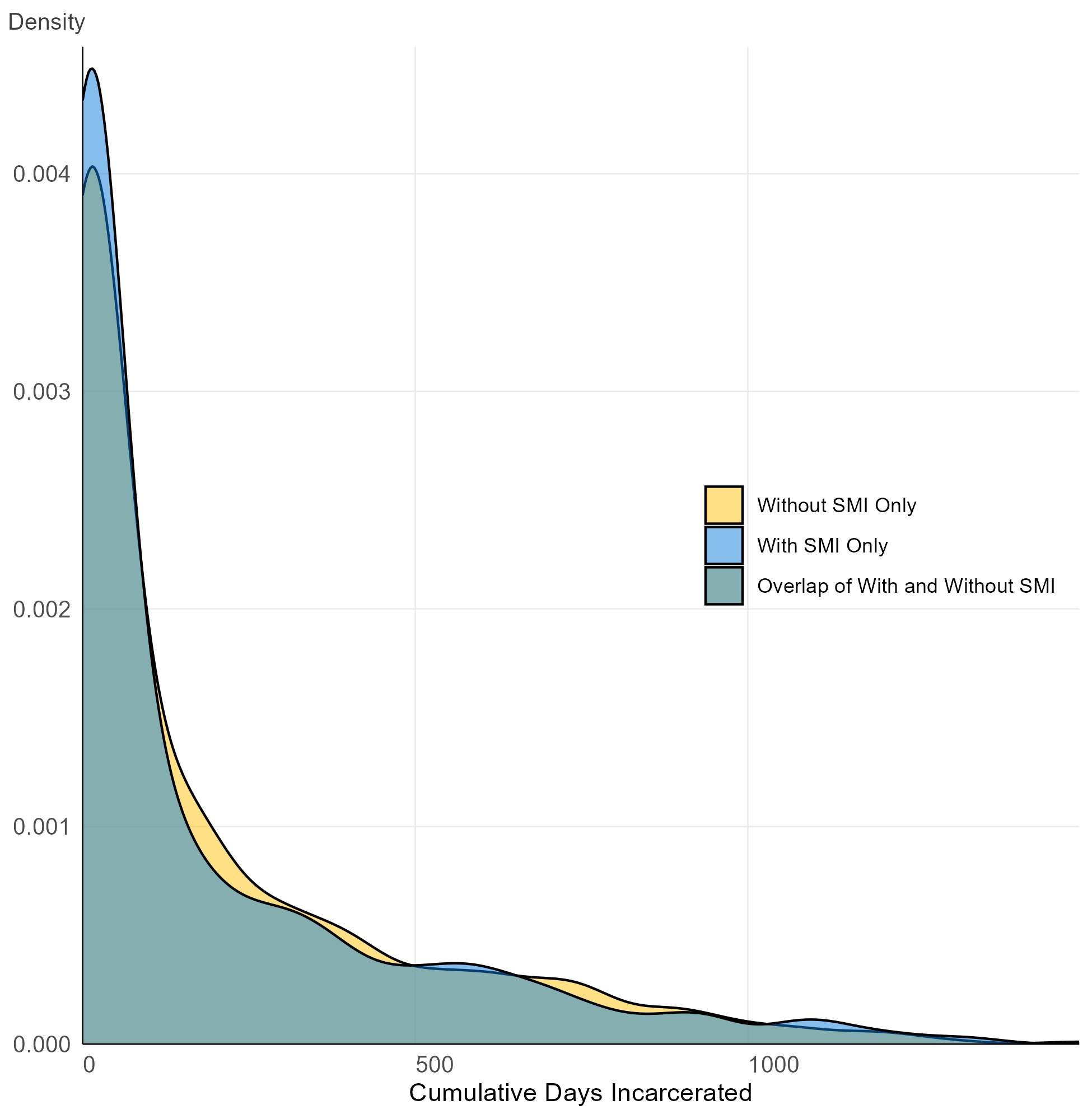


Density plot showing the distribution of total cumulative jail days for all incarcerations during the study period, stratified by serious mental illness status. Individuals with no prior booking history were excluded. Abbreviations: SMI, Serious Mental Illness
