# Supplementary material for: Unveiling Intersecting Experiences: Investigating Health Care and Jail System Interaction Before and After Incarceration Among Adults with Serious Mental Illness in San Francisco
Source: J Urban Health. 2026 Feb 24;103(3):533–41. doi: 10.1007/s11524-026-01058-2 (PMC13315379; doi:10.1007/s11524-026-01058-2)
Supplement: Supplementary file 2 — (DOCX 76.2 KB) [file 11524_2026_1058_MOESM2_ESM.docx]

**Supplemental Fig. 2 Incarceration Period: Distribution of Total Jail Bookings by Serious Mental Illness Status**

**
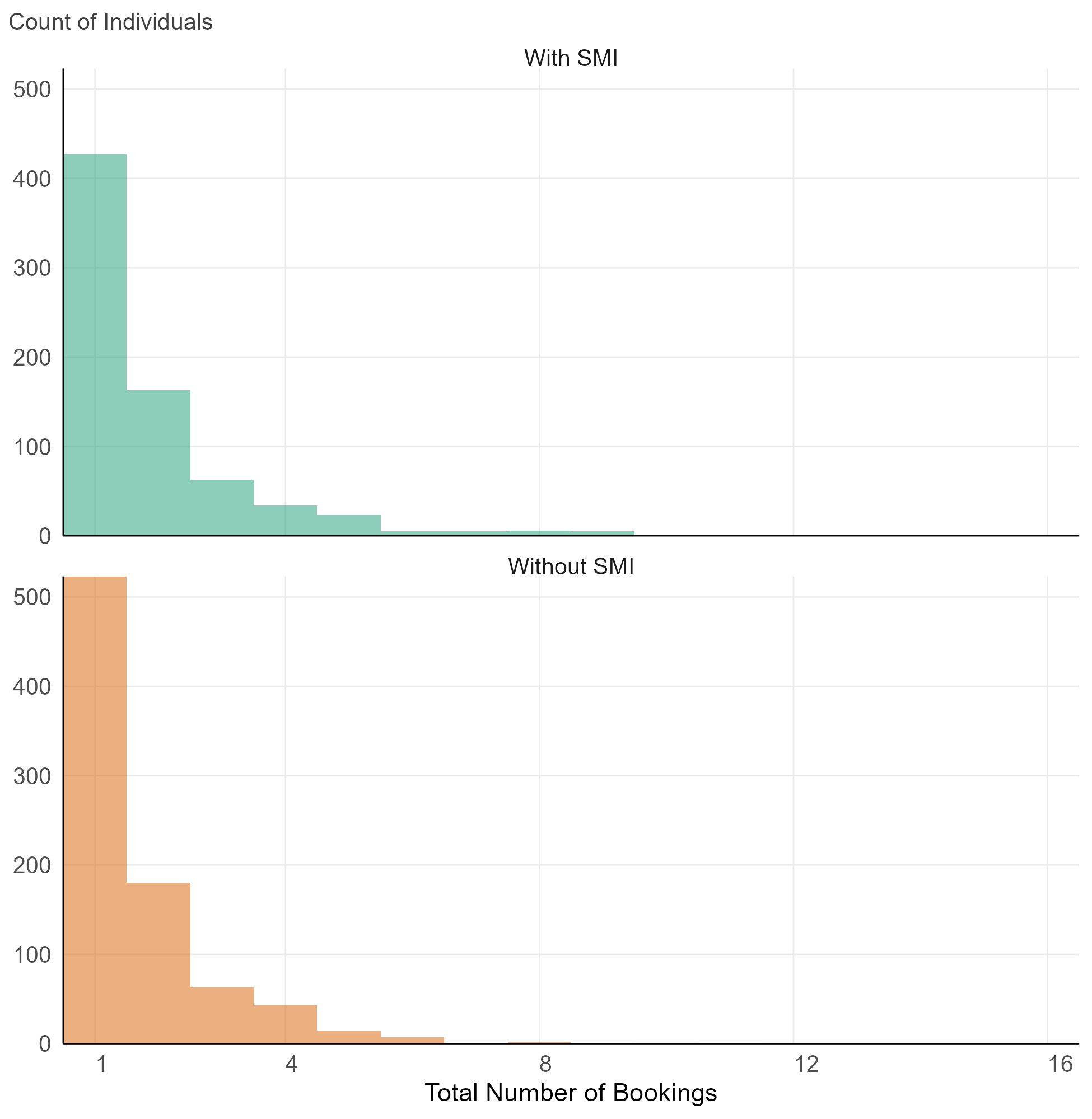
**

Histograms showing the distribution of total jail bookings per individual during the incarceration period, stratified by serious mental illness status. Abbreviations: SMI, Serious Mental Illness
