# Supplementary material for: Unveiling Intersecting Experiences: Investigating Health Care and Jail System Interaction Before and After Incarceration Among Adults with Serious Mental Illness in San Francisco
Source: J Urban Health. 2026 Feb 24;103(3):533–41. doi: 10.1007/s11524-026-01058-2 (PMC13315379; doi:10.1007/s11524-026-01058-2)
Supplement: Supplementary file 3 — (DOCX 179 KB) [file 11524_2026_1058_MOESM3_ESM.docx]

**Supplemental Fig. 3 Incarceration Period: Distribution of Cumulative Jail Days for all Jail Incarcerations by Serious Mental Illness Status.**


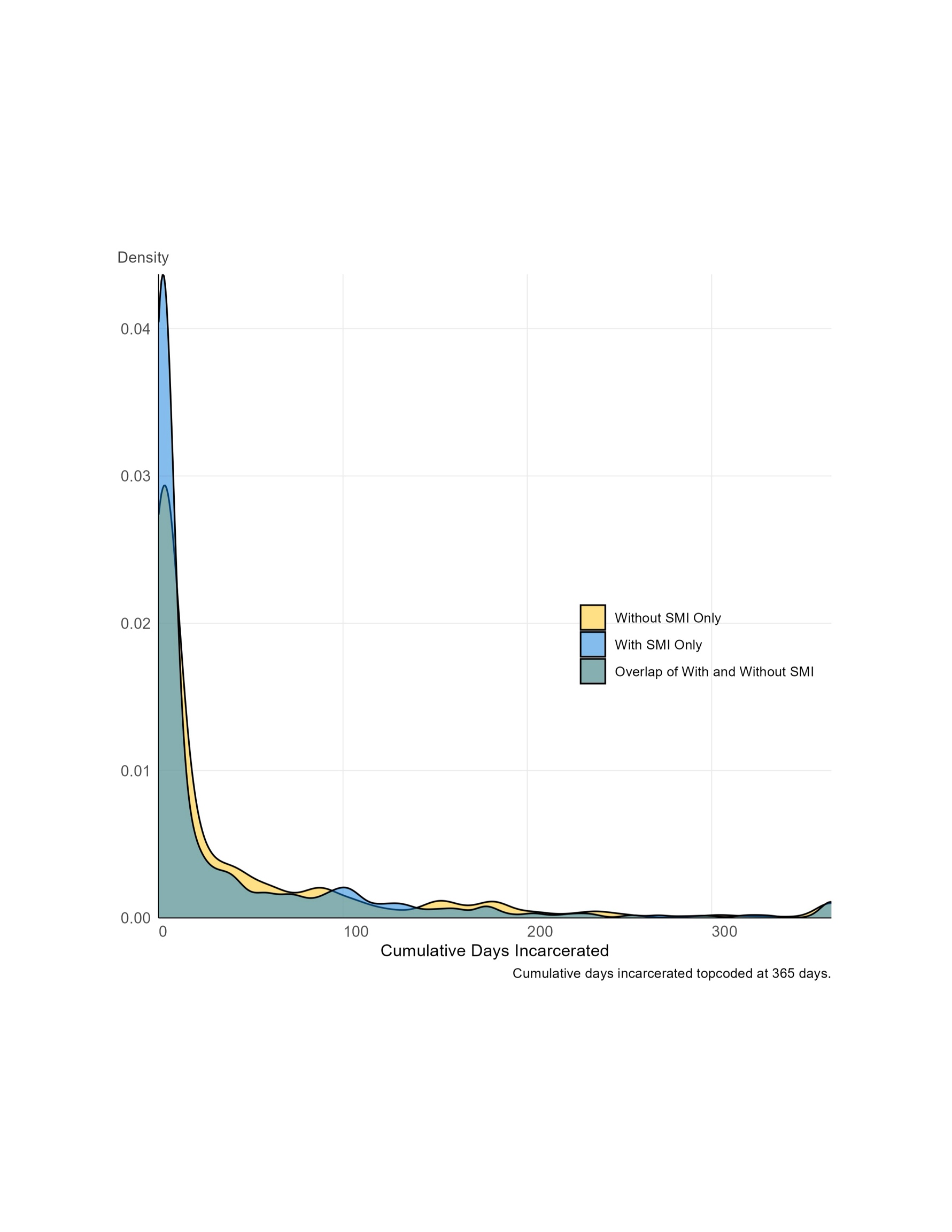


Density plot showing the distribution of total cumulative jail days for all incarcerations during the study period, stratified by serious mental illness status. Cumulative days incarcerated top-coded at 365 days. Abbreviations: SMI, Serious Mental Illness
