# Supplementary material for: Unveiling Intersecting Experiences: Investigating Health Care and Jail System Interaction Before and After Incarceration Among Adults with Serious Mental Illness in San Francisco
Source: J Urban Health. 2026 Feb 24;103(3):533–41. doi: 10.1007/s11524-026-01058-2 (PMC13315379; doi:10.1007/s11524-026-01058-2)
Supplement: Supplementary file 5 — (DOCX 27.7 KB) [file 11524_2026_1058_MOESM5_ESM.docx]

| **Supplemental Table III. Incarceration Period: Regression Model of the Longest Jail Stay (Units are in Days)** | |
| --- | --- |
| **Variable** | **Estimate (95% CI)** |
| **Has SMI** | 11.09* (1.05, 21.13) |
| **Age (Reference: 18–24)** |  |
| Age 25–34 | -21.52* (-42.70, -0.35) |
| Age 35–44 | -23.90* (-45.10, -2.71) |
| Age 45–54 | -14.98 (-36.60, 6.64) |
| Age 55–64 | -22.77 (-46.88, 1.34) |
| Age 65+ | -34.69 (-76.05, 6.68) |
| **Race & Ethnicity (Reference: White)** |  |
| Asian or Pacific Islander | 6.83 (-18.35, 32.00) |
| Black | 11.47 (-0.06, 22.99) |
| Latinx | -4.60 (-20.42, 11.22) |
| Multi-racial and Other^a^ | 5.09 (-21.21, 31.40) |
| **Gender (Reference: Woman)** |  |
| Male | 17.47** (5.31, 29.63) |

Significance levels: *p < 0.05; **p < 0.01; ***p < 0.001

^a^The Multiracial and Other category was combined due to small numbers among individuals self-identifying as Native American or Indigenous, Multiracial, and those not reporting a race or ethnicity.

Abbreviations: SMI, Serious Mental Illness
