# Supplementary material for: Unveiling Intersecting Experiences: Investigating Health Care and Jail System Interaction Before and After Incarceration Among Adults with Serious Mental Illness in San Francisco
Source: J Urban Health. 2026 Feb 24;103(3):533–41. doi: 10.1007/s11524-026-01058-2 (PMC13315379; doi:10.1007/s11524-026-01058-2)
Supplement: Supplementary file 6 — (DOCX 13.2 KB) [file 11524_2026_1058_MOESM6_ESM.docx]

**Supplementary Table I. Catalog of Urgent and Emergent Services Captured by the San Francisco Department of Public Health’s Coordinated Care Management System**

| **System** | **Urgent/Emergent Service** | **Unit of Measure** |
| --- | --- | --- |
| **Physical Health System** | Emergency Department | Visit |
|  | Hospital Medical Inpatient | Stay |
|  | Urgent Care Clinic | Visit |
| **Mental Health System** | Psychiatric Emergency Services | Visit |
|  | Hospital Psychiatric Inpatient | Stay |
|  | Psychiatric Urgent Care Clinic | Visit |
| **Substance Use Disorder System** | Medical Detoxification | Stay |
|  | Social Detoxification | Stay |
|  | Emergency Department | Visit |
