# Supplementary material for: Unveiling Intersecting Experiences: Investigating Health Care and Jail System Interaction Before and After Incarceration Among Adults with Serious Mental Illness in San Francisco
Source: J Urban Health. 2026 Feb 24;103(3):533–41. doi: 10.1007/s11524-026-01058-2 (PMC13315379; doi:10.1007/s11524-026-01058-2)
Supplement: Supplementary file 7 — (DOCX 12.8 KB) [file 11524_2026_1058_MOESM7_ESM.docx]

**Supplementary Table II. List of Elixhauser Comorbidities Used to Calculate Elixhauser Physical Health Comorbidity Score**

| **Elixhauser Comorbidities** |
| --- |
| AIDS/HIV, blood loss anemia, cardiac arrhythmias, chronic pulmonary disease, coagulopathy, congestive heart failure, deficiency anemia, complicated diabetes, uncomplicated diabetes, fluid and electrolyte disorders, complicated hypertension, uncomplicated hypertension, hypothyroidism, liver disease, lymphoma, metastatic cancer, obesity, other neurological disorders, paralysis, peptic ulcer disease excluding bleeding, peripheral vascular disease, pulmonary circulation disorder, renal failure, rheumatic arthritis and collagen vascular disease, solid tumor without metastasis, valvular disease, weight loss |
